# Berberine attenuates fructose-induced insulin resistance by stimulating the hepatic LKB1/AMPK/PGC1α pathway in mice

**DOI:** 10.1080/13880209.2020.1756349

**Published:** 2020-05-12

**Authors:** Yucheng Li, Baoying Wang, Jiduo Shen, Ming Bai, Erping Xu

**Affiliations:** a Henan Key Laboratory for Modern Research on Zhongjing’s Herbal Formulae, Scientific Research and Experiment Center, Henan University of Chinese Medicine, Zhengzhou, Henan, PR China; b College of Pharmacy, Henan University of Chinese Medicine, Zhengzhou, Henan, PR China

**Keywords:** Insulin sensitivity, metabolic homeostasis, alkaloid, signalling pathway

## Abstract

**Context:**

Berberine is an alkaloid that possesses various pharmacologic effects.

**Objective:**

To explore the mechanism of berberine to improve insulin sensitivity in fructose-fed mice.

**Materials and methods:**

Sixty male ICR mice were randomly divided into 6 groups (10 mice in each group): control, fructose, pioglitazone (10 mg/kg) and berberine (50, 100, and 200 mg/kg). Except for the control group, the mice received 20% fructose drinking for 10 weeks. Pioglitazone and berberine were orally administered once daily during the last 4 weeks. The insulin sensitivity was evaluated using an oral glucose tolerance test (OGTT). The serum levels of fasting glucose and insulin, blood lipids, and hormones were determined. The hepatic AMP and ATP contents were detected using high performance liquid chromatography (HPLC) analysis, and the protein expression was examined by immunoblotting.

**Results:**

Berberine significantly reversed the insulin resistance induced by fructose, including lowering fasting insulin levels (from 113.9 to 67.4) and area under the curve (AUC) during OGTT (from 1310 to 1073), decreasing serum leptin (from 0.28 to 0.13) and increasing serum adiponectin levels (from 1.50 to 2.80). Moreover, berberine enhanced the phosphorylation levels of protein kinase B (PKB/AKT; 2.27-fold) and glycogen synthase kinase-3β (GSK3β; 2.56-fold), and increased hepatic glycogen content (from 0.19 to 1.65). Furthermore, berberine upregulated the protein expression of peroxisome proliferator activated receptor gamma coactivator 1α (PGC1α; 2.61-fold), phospho-AMP-activated protein kinase (p-AMPK; 1.35-fold) and phospho-liver kinase B1 (p-LKB1; 1.41-fold), whereas it decreased the AMP/ATP ratio (from 4.25 to 1.82).

**Conclusion:**

The present study demonstrated the protective effects of berberine against insulin resistance induced by fructose. Our findings may provide an experimental basis for the application of berberine in the treatment of insulin resistance.

## Introduction

Insulin resistance is a clinical pathological condition characterized by glucose intolerance and impaired insulin sensitivity (Lebovitz 2001). Insulin resistance is closely related to various metabolic disorders, including obesity, metabolic syndrome, and fatty liver (Gluvic et al. 2017; Watt et al. 2019). Importantly, insulin resistance is commonly considered as the core feature of type 2 diabetes (T2D; Kahn et al. 2006). Restoring insulin sensitivity and improving metabolic homeostasis is crucial in treatment of metabolic disorders. Thiazolidinediones, known as insulin sensitizers, are drugs commonly used to improve insulin sensitivity in the treatment of T2D. However, the various and serious side effects limit their clinical application. For example, rosiglitazone increases the risk of heart disease (Singh et al. 2007), and troglitazone results in serious liver injury (Neuschwander-Tetri et al. 1998). Therefore, searching for an alternative insulin sensitizer is an important strategy for treating metabolic disorders.

It is well documented that AMP-activated protein kinase (AMPK) is regarded as an important target in the treatment of diabetes or metabolic syndrome (Carling 2004). AMPK is activated through its interaction with AMP and phosphorylation by upstream kinases (Woods et al. 2003; Carling 2004). Activated AMPK regulates a variety of energy metabolic processes by switching on ATP-generating pathways such as fatty acid oxidation and glycolysis and by switching off ATP-consuming pathways such as synthesis of fatty acids and cholesterol (Minokoshi et al. 2002). Moreover, AMPK is strongly beneficial for enhancing insulin sensitivity by many pathways (Kola et al. 2006; Ruderman et al. 2013). Of interest, previous studies have revealed that antidiabetic drugs, such as metformin and thiazolidinediones, can significantly activate AMPK signalling (Bajaj et al. 2003; Lin et al. 2017). Therefore, the activation of AMPK has been proposed as an important target for improving insulin sensitivity (Kim et al. 2009; Xin et al. 2016).

Berberine, well known as an AMPK activator (Wang et al. 2018), is an alkaloid that possesses various pharmacologic effects, including reducing blood glucose, enhancing insulin sensitivity and improving hyperlipidaemia (Cheng et al. 2006; Kim et al. 2009; Zhao et al. 2017). In recent years, the hypothesis that berberine activates AMPK by inhibiting ATP production in the mitochondrial respiratory chain has been widely accepted (Turner et al. 2008; Hou et al. 2018). It is well known that AMP promotes allosteric changes of AMPK that makes it more easily phosphorylated by upstream kinases, such as liver kinase B1 (LKB1) and Ca^2+^/calmodulin-dependent protein kinase kinase beta (CaMKKβ; Sriwijitkamol et al. 2006; Willows et al. 2017). Therefore, whether improving insulin sensitivity by berberine involves upstream kinases of AMPK remains unclear. The present study aimed to investigate the effects of berberine on insulin resistance, and to clarify the underlying mechanism of berberine enhancing insulin sensitivity and restoring metabolic homeostasis by activating AMPK signalling.

## Materials and methods

### Reagents and drugs

Pioglitazone hydrochloride was purchased from Deyuan Pharmaceutical Co., Ltd. (Lianyungang, Jiangsu Province, China). d-Fructose was supplied by Xiwang Food Co., Ltd. (Jinan, Shandong Province, China). Berberine (98% HPLC grade purity) was purchased from Aladdin Co., Ltd. (Shanghai, China). The standard references of AMP and ATP (99% purity) were purchased from Sigma-Aldrich (St Louis, MO, USA). The primary antibodies against p-AKT (Ser473), AKT, p-AMPK (Thr172), AMPK, p-LKB1 (Ser428), LKB1, p-GSK3β (Ser9), GSK3β and PGC1α were from Cell Signalling Technology (Danvers, MA, USA). Antibody against CaMKKβ was from Proteintech (Wuhan, Hubei Province, China). Antibodies against GAPDH and HRP-conjugated IgG were from Kangcheng (Shanghai, China).

### Animals

Male ICR mice (18 ∼ 22 g) were purchased from Hunan Slac Animal Centre (Changsha, Hunan Province, China). Mice were maintained in controlled environment (22 ∼ 25 °C temperature and 40 ∼ 60% humidity) with a 12 h light–dark cycle and were given *ad libitum* access to food and water. After adaption for 1 week, all mice were randomly divided into a control group (*n* = 10) and a fructose group (*n* = 50). The control group received tap water and the fructose group received 20% fructose for drinking. After 6 weeks, the fructose group was randomly divided into 5 subgroups (*n* = 10): fructose group (normal saline), pioglitazone group (10 mg/kg) and berberine groups (50, 100, 200 mg/kg). The drugs were suspended in normal saline. Each group was dosed by oral gavage once daily for 4 weeks. Body weight and food intake were recorded weekly. All animal experimental procedures were approved by the Committee of Animal Care of Henan University of Chinese Medicine (DWLL-201402009).

### Oral glucose tolerance testing

Oral glucose tolerance testing (OGTT) was carried out as described in our previous study (Li et al. 2016). Followed a 14 h fast, mice were administered a 50% glucose solution (1.5 g/kg) orally. Then, the blood glucose was determined at 0, 30, 60, 90, and 120 min after administration by an ACCU-CHEK blood glucose metre (Roche Diagnostics Co., Ltd, Shanghai, China).

### Blood and tissue collection

Forty-eight hours after the OGTT, blood was obtained from a vein of the eyeball, and the serum was separated by centrifugation at 4000 *g* for 10 min. Then, all mice were killed by cervical dislocation. The liver was divided into several small pieces that were immediately frozen in liquid nitrogen and stored at −80 °C for subsequent analysis.

### Biochemical analysis

The serum glucose, triglyceride (TG), total cholesterol (TC), low-density lipoprotein cholesterol (LDL-C) and high-density lipoprotein cholesterol (HDL-C) were measured by an AU400 automatic biochemical analyser (OLYMPUS, Tokyo, Japan). The insulin, leptin, orexin, adiponectin and glucagon levels were determined by commercial enzyme-linked immunosorbent assay kits according to the manufacturer’s instructions (CUSABIO, Wuhan, China).

### Measurement of hepatic TG and glycogen content

As described in our previous report (Li et al. 2018), we extracted the hepatic lipids for TG determination according to the method of Folch with some modifications (Folch et al. 1957). Briefly, the liver was homogenized in a 20-fold volume of a chloroform/methanol (2:1) mixture. After shaking for 15 ∼ 20 min, the homogenate was centrifuged at 2000 *g* for 10 min. The supernatants were transferred to a new tube, and 0.2-fold volume of water was added to the tube. Following centrifugation at 2000 *g* for 10 min, the lower layer (chloroform phase) was collected for TG determination. For hepatic glycogen determination, the mixture of liver tissue and a 3-fold volume of alkali solution were placed in boiling water for 20 min. After centrifugation at 2000 *g* for 10 min, the extract was used for the determination of the glycogen content. The determination methods for TG and glycogen were performed following the instructions of the kits (Jiancheng Institute of Bioengineering, Nanjing, China).

### Measurement of hepatic AMP and ATP content

Liver tissue (approximately 300 mg) was homogenized in a 10-fold volume of cold 0.4 M perchloric acid. After centrifugation at 2000 *g* for 10 min at 4 °C, the supernatants were transferred to another tube and mixed with an equal volume of 1 M KH_2_PO_4_. The pH was adjusted to 6.5 with 1 M KOH. The liquid was centrifuged at 10,000 *g* for 15 min at 4 °C. After filtration with 0.45 μm membrane filter, the samples were stored at −80 °C until analysis. For AMP/ATP ratio determination, a Waters 2695 Alliance HPLC equipped with a Hypersil C18 column (250 mm × 4.6 mm, 5 μm) was used. The conditions were as follows: sample injection, 20 μL; flow rate, 1 mL/min; wave length, 254 nm. A linear gradient consisting of 0.05 M KH_2_PO_4_ adjusted to pH 6.5 containing 40% of 5 mM tetrabutylammonium hydroxide was used as the initial eluent and was increased to 30% (v/v) methanol over a period of 30 min.

### Western blotting

The frozen liver sample was homogenized in a 10-fold volume RIPA lysate containing cOmplete™ ULTRA protease inhibitors (Roche, Shanghai, China) and PhosSTop™ phosphatase inhibitor cocktail (Roche, Shanghai, China). After centrifuging at 12,000 *g* for 20 min, the supernatants were collected for protein concentration determination by a BCA kit (CWBIO, Beijing, China). Total protein was separated by SDS-PAGE and transferred to PVDF membranes (Millipore, Shanghai, China). After 2 h of blocking by 5% non-fat milk (BD Biosciences, Franklin Lakes, NJ, USA), the membranes were incubated with primary antibodies (PGC1α, 1:1000; p-GSK3β, 1:1000; GSK3β, 1:1000; p-AKT, 1:1000; AKT, 1:1000; AMPK, 1:1000; p-AMPK, 1:500; LKB1, 1:1000; p-LKB1, 1:500; CaMKKβ, 1:500; GAPDH, 1:5000) overnight at 4 °C. After washing with TBST 3 times, the HRP-conjugated secondary antibody (1:60,000) was added to the membranes for 1 h at room temperature. ECL reagents (Merck, Darmstadt, Germany) and X-ray film (Kodak, Rochester, NY, USA) were used to visualize the immunoblotting bands. The integrated optical density (IOD) was analysed by Image-Pro Plus 6.0 software (Media Cybernetics, Silver Spring, MD, USA). The relative protein expression was normalized using GAPDH as an internal reference.

### Statistical analysis

All data are expressed as the mean ± S.E.M. One-way ANOVA followed by Dunnett’s *post hoc* test was used for statistical analysis (SPSS20.0 statistical package, SPSS Inc., Chicago, IL, USA). Differences were considered statistically significant at *p* < 0.05.

## Results

### Body weight, food consumption, abdominal fat and liver index

**Figure 1.**
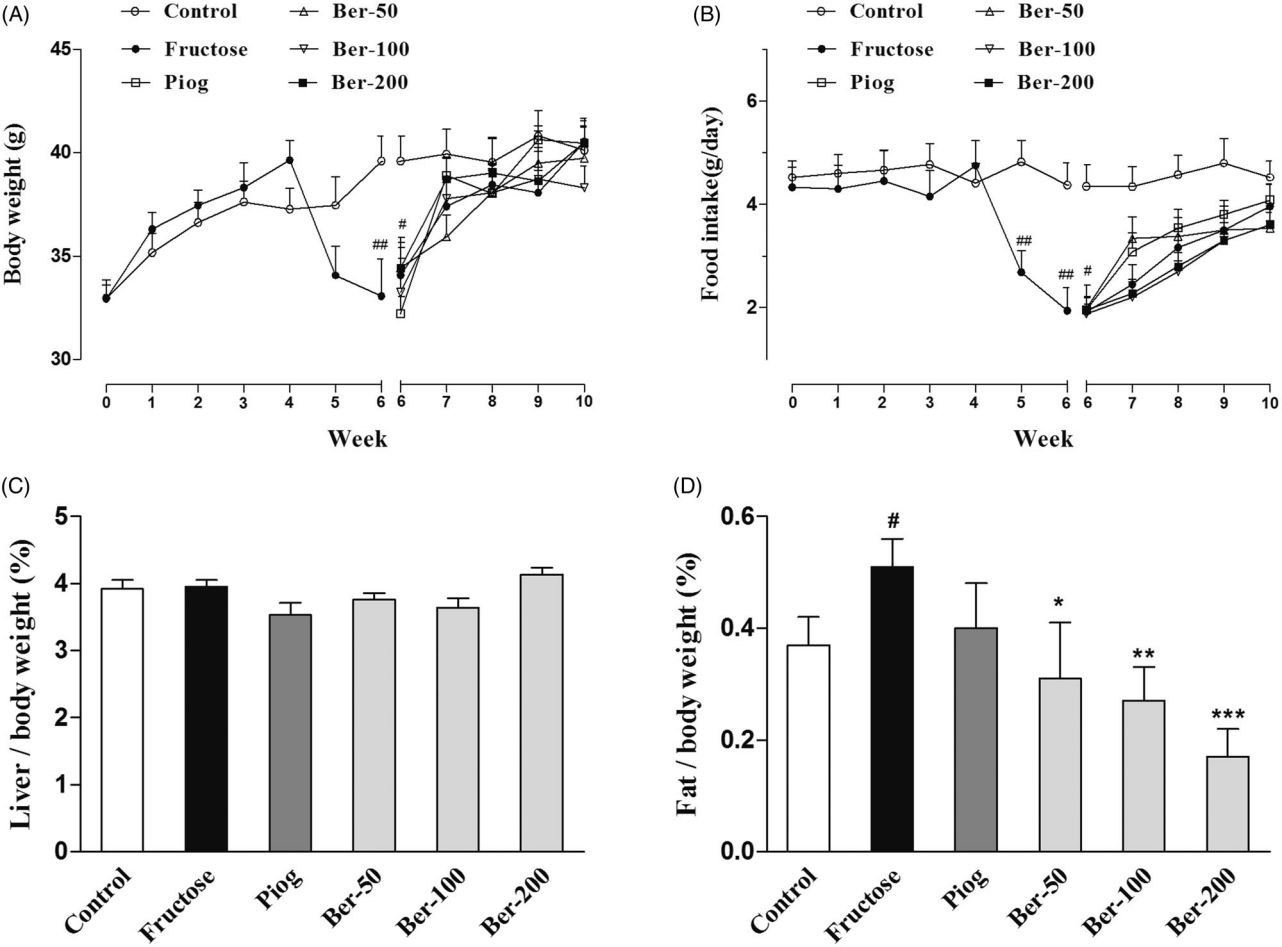
Effects of berberine on body weight, food intake, abdominal fat and liver organ index in fructose-fed mice. (A) Body weight; (B) food intake; (C) liver index; (D) abdominal fat index. Data were expressed as the mean ± S.E.M. (*n* = 10). #*p* < 0.05, ##*p* < 0.01 vs. control group. *p < 0.05, **p < 0.01, ***p < 0.001 vs. fructose group.

As shown in Figure 1(A,B), the body weight of the fructose-fed mice was reduced between week 5 and week 6 (week 6: *p* < 0.05). There was no significant difference in all groups after week 7. The food intake was also significantly reduced in the fructose group from week 5 to week 6 (week 5: *p* < 0.01; week 6: *p* < 0.01). Berberine and pioglitazone did not change the body weight or food intake in the fructose-fed mice.

In addition, the ratio of abdominal fat to body weight in the fructose group was significantly higher than that of the control (*p* < 0.05). Berberine significantly reduced the level of abdominal fat (50 mg/kg: *p* < 0.05; 100 mg/kg: *p* < 0.01; 200 mg/kg: *p* < 0.001), whereas pioglitazone had no effect on the abdominal fat and liver index (Figure 1(C,D)).

### Insulin sensitivity and serum levels of lipids and hormones

**Figure 2.**
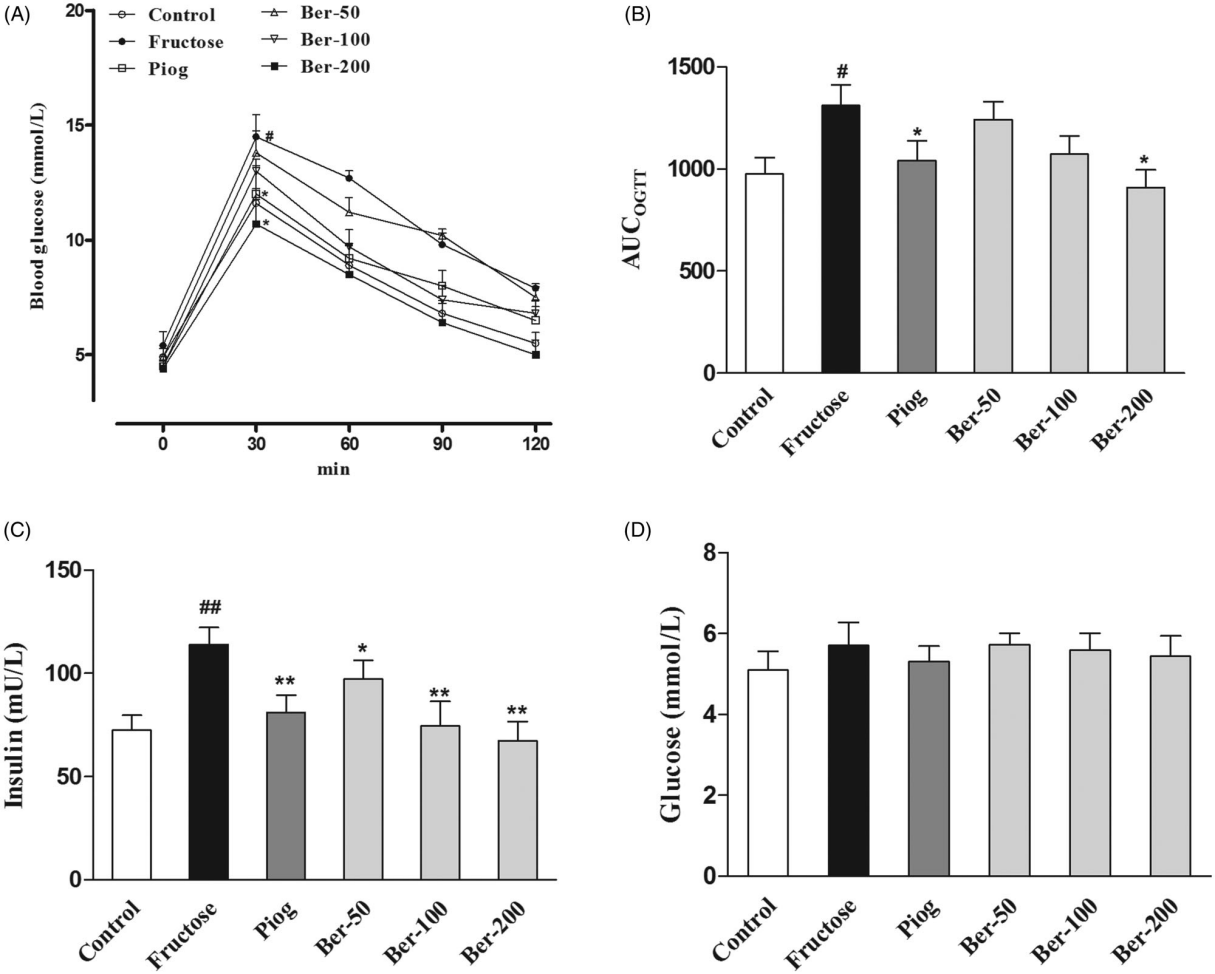
Effects of berberine on insulin resistance in fructose-fed mice. (A) OGTT; (B) AUCOGTT; (C) fasting serum insulin; (D) fasting serum glucose. Data were expressed as the mean ± S.E.M. (*n* = 10). #*p* < 0.05, ##*p* < 0.01 vs. control group. *p < 0.05, **p < 0.01 vs. fructose group.

Berberine and pioglitazone significantly decreased the serum fasting insulin level (*p* < 0.01), and alleviated insulin resistance as shown by the oral glucose tolerance test (*p* < 0.05) in fructose-fed mice without affecting fasting glucose levels (Figure 2). The serum levels of TG, TC, HDL-C and LDL-C in the fructose group were significantly higher than in the control group (*p* < 0.05). Berberine and pioglitazone significantly reversed the dyslipidemia induced by fructose (*p* < 0.05; Table 1). In addition, berberine reduced the serum levels of leptin (*p* < 0.05) and orexin (*p* < 0.05), while it increased the serum adiponectin level in fructose-fed mice (*p* < 0.001). Both berberine and pioglitazone failed to alter the serum level of glucagon (Table 2).

**Table 1.** The effects of berberine on serum lipids profiles in fructose-fed mice.

Group	Dose (mg/kg)	TG (mmol/L)	TC (mmol/L)	HDL-C (mmol/L)	LDL-C (mmol/L)
Control	–	0.88 ± 0.04	3.92 ± 0.28	2.61 ± 0.19	0.56 ± 0.05
Fructose	–	1.13 ± 0.09^#^	4.97 ± 0.39^#^	3.27 ± 0.24^#^	0.70 ± 0.04^#^
Pioglitazone	10	1.03 ± 0.09	3.32 ± 0.32**	2.37 ± 0.23*	0.41 ± 0.04***
Berberine	50	0.77 ± 0.09*	4.37 ± 0.23*	2.32 ± 0.27*	0.67 ± 0.08
	100	0.63 ± 0.03***	3.93 ± 0.41	2.46 ± 0.21*	0.72 ± 0.06
	200	0.62 ± 0.04***	3.92 ± 0.15*	2.55 ± 0.18*	0.50 ± 0.04**

Data were expressed as mean ± S.E.M. *n* = 10. ^#^*p* < 0.05 vs. control group; *p < 0.05, **p < 0.01, ***p < 0.001 vs. fructose group.

**Table 2.** The effects of berberine on serum hormones level in fructose-fed mice.

Group	Dose (mg/kg)	Orexin (pg/mL)	Leptin (ng/mL)	Adiponectin (ng/mL)	Glucagon (ng/L)
Control	–	15.69 ± 2.60	0.13 ± 0.03	2.68 ± 0.20	194.1 ± 7.44
Fructose	–	39.40 ± 5.05^#^	0.28 ± 0.06^#^	1.50 ± 0.08^###^	245.6 ± 17.69
Pioglitazone	10	28.20 ± 2.77	0.29 ± 0.05	2.33 ± 0.14***	227.7 ± 9.27
Berberine	50	30.36 ± 3.61	0.16 ± 0.05*	2.35 ± 0.09***	225.5 ± 21.43
	100	23.45 ± 2.63*	0.16 ± 0.02*	2.46 ± 0.24***	206.9 ± 13.64
	200	20.18 ± 4.30*	0.13 ± 0.03*	2.80 ± 0.18***	197.1 ± 18.27

Data were expressed as mean ± S.E.M. *n* = 10. ^#^*p* < 0.05, ^###^*p* < 0.001 *vs* control group; *p < 0.05, **p < 0.01, ***p < 0.001 *vs* fructose group.

### The hepatic TG and glycogen content

**Figure 3.**
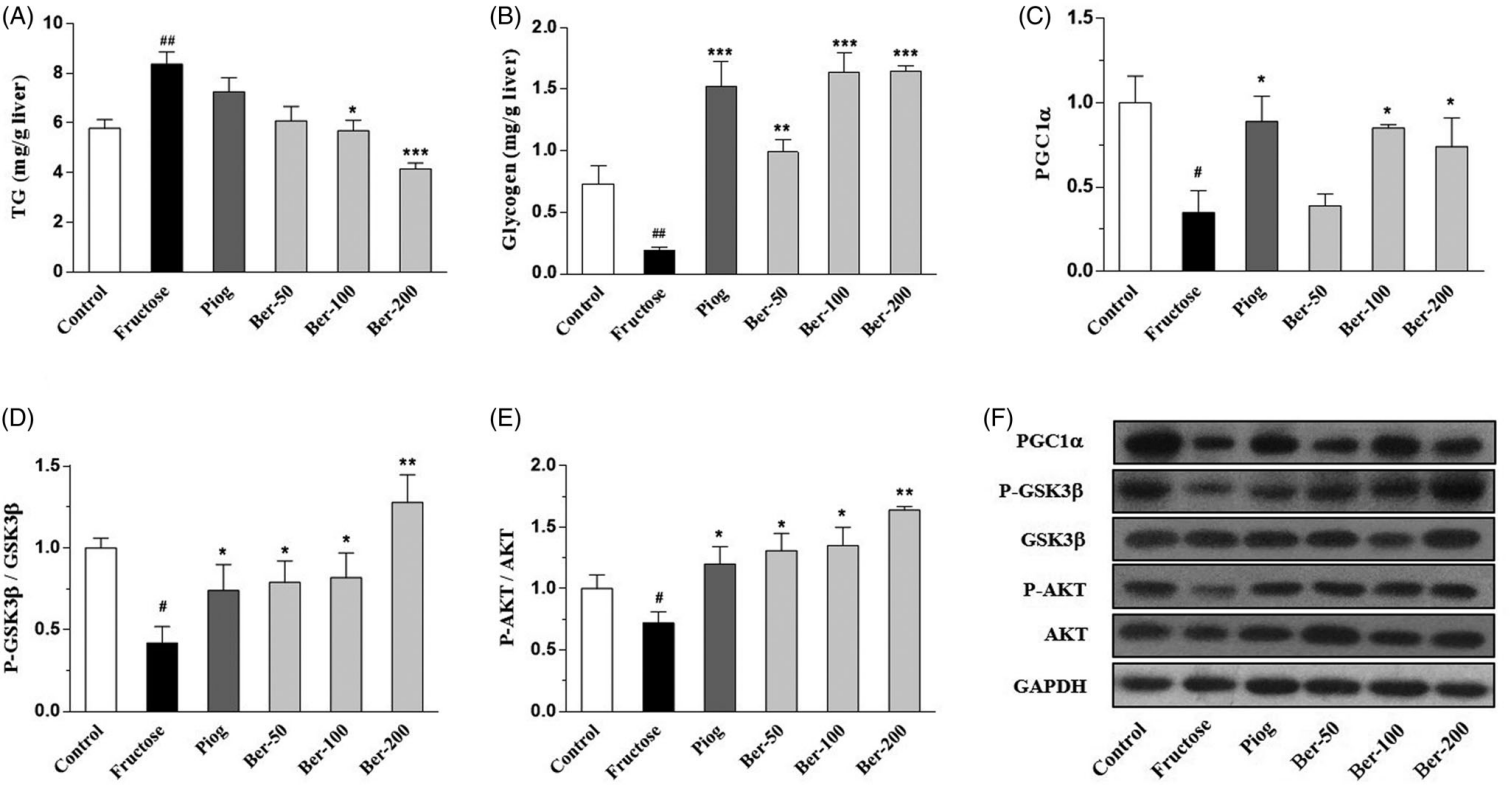
The effects of berberine on hepatic TG and glycogen content in fructose-fed mice. (A) Hepatic TG content; (B) hepatic glycogen content; (C) the protein expression of PGC1? in liver; (D) The protein expression of GSK? in liver; (E) the protein expression of AKT in liver; (F) the immunoblot bands. Data were expressed as the mean ± S.E.M. (*n* = 10 for TG and glycogen; *n* = 6 for WB). #*p* < 0.05, ##*p* < 0.01 vs. control group. *p < 0.05, **p < 0.01, ***p < 0.001 vs. fructose group.

As shown in Figure 3, fructose caused a significant increase in TG content (*p* < 0.01) and a decrease in glycogen content in the liver (*p* < 0.01). These changes were reversed by berberine (*p* < 0.05). Pioglitazone increased the hepatic glycogen content (*p* < 0.001) without affecting TG. To investigate the molecular mechanism of berberine increasing hepatic glycogen and decreasing TG, we examined the protein expression of PGC1α, GSK3β and AKT. The WB results displayed a significant reduction of PGC1α and the phosphorylation levels of GSK3β and AKT in fructose-fed mice (*p* < 0.05), while berberine and pioglitazone reversed these changes (*p* < 0.05).

### AMPK signalling

**Figure 4.**
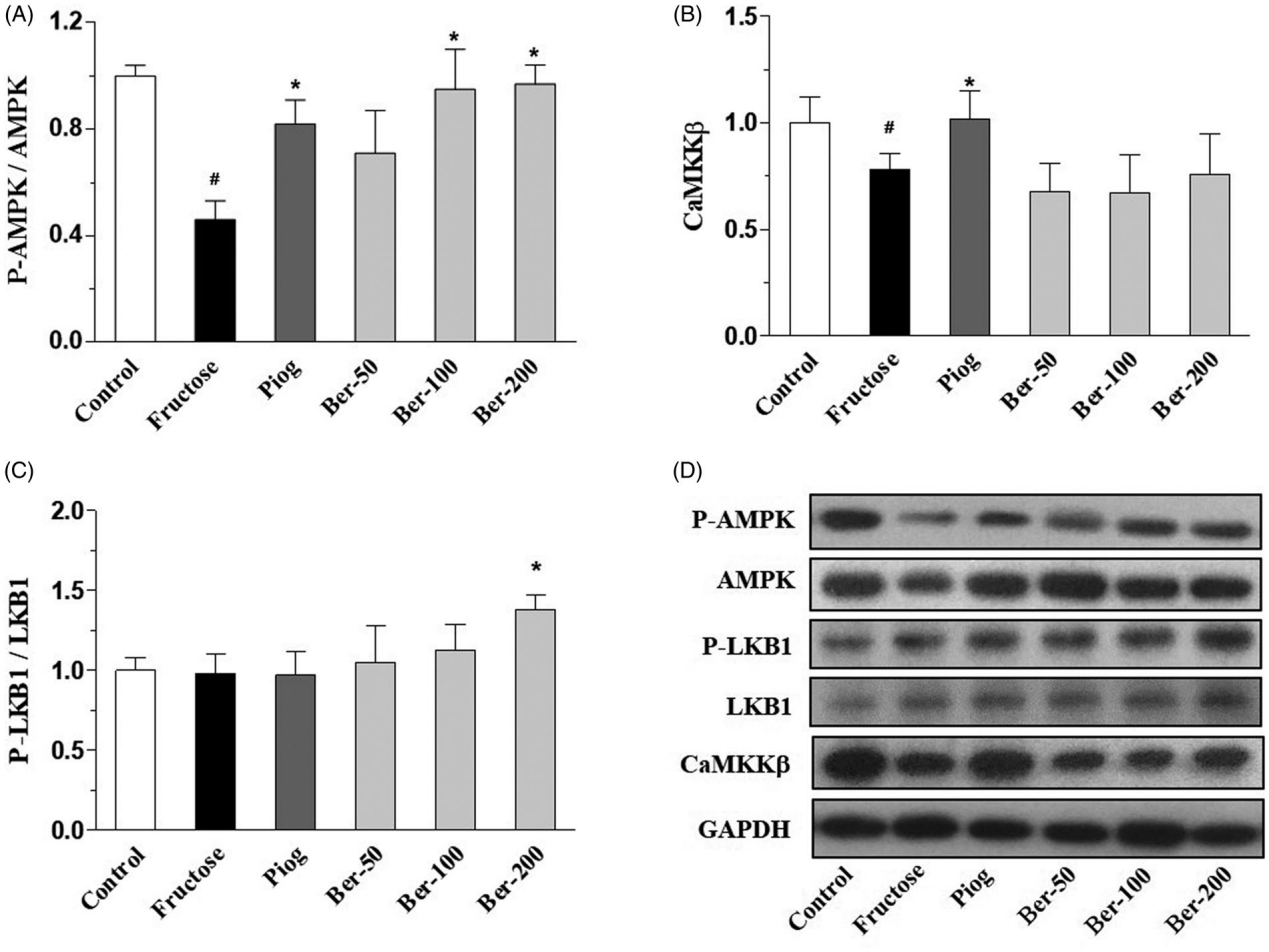
The effects of berberine on hepatic AMPK signalling in fructose-fed mice. (A) The protein expression of AMPK; (B) the protein expression of CaMKKβ; (C) the protein expression of LKB1; (D) the immunoblot bands. Data were expressed as the mean ± S.E.M. (n = 6). #*p* < 0.05 vs. control group. *p < 0.05 vs. fructose group.

**Figure 5.**
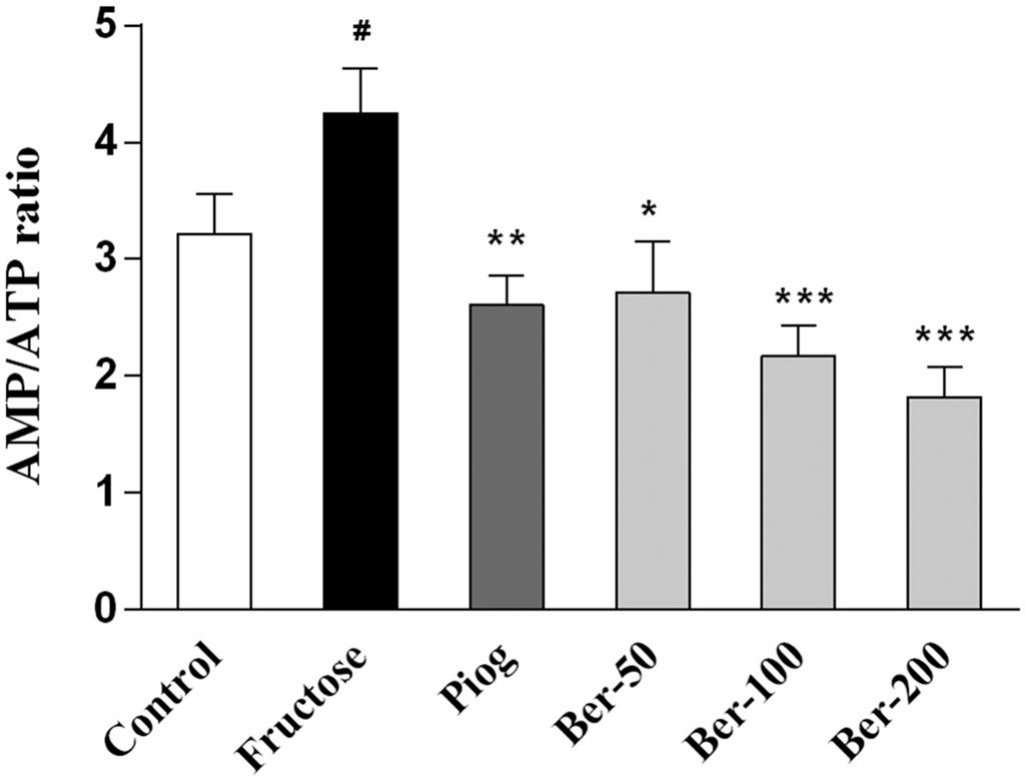
The effects of berberine on hepatic AMP/ATP ratio in fructose-fed mice. Data were expressed as the mean ± S.E.M. (*n* = 10). #*p* < 0.05 vs. control group. *p < 0.05, **p < 0.01, ***p < 0.001 vs. fructose group.

As shown in Figures 4 and 5, fructose significantly decreased the phosphorylation levels of AMPK (*p* < 0.05), downregulated the protein expression of CaMKKβ (*p* < 0.05), and increased the AMP/ATP ratio (*p* < 0.05). Both berberine (200 mg/kg) and pioglitazone upregulated the expression of p-AMPK (*p* < 0.05) and reduced the AMP/ATP ratio (*p* < 0.05). Moreover, berberine significantly increased the phosphorylation levels of LKB1 (*p* < 0.05), whereas pioglitazone increased CaMKKβ expression in the fructose group (*p* < 0.05).

## Discussion

Insulin resistance is a core symptom among various metabolic related disorders, including metabolic syndrome, T2D, obesity and non-alcoholic fatty liver disease. Previous reports revealed that berberine can improve insulin resistance by activating AMPK signalling (Chang et al. 2013). However, the underlying mechanism remains unclear. The present study verified the protective effects of berberine against fructose-induced insulin resistance and metabolic abnormalities and suggested these effects might be mediated by the LKB1/AMPK/PGC1α pathway.

Numerous researches have confirmed that chronic fructose consumption impairs insulin sensitivity and induces insulin resistance (Elliott et al. 2002; Deer et al. 2015; Ter Horst et al. 2016). Therefore, fructose has been widely used to establish an experimental animal model of insulin resistance (Elliott et al. 2002; Mamikutty et al. 2014; Ter Horst et al. 2016). In the present study, we evaluated the effects of berberine on insulin sensitivity and its metabolic consequences in fructose-fed mice. Our results showed that chronic fructose drinking resulted in insulin resistance, which was evidenced by a markedly elevated serum insulin level and area under the curve (AUC) during OGTT. Moreover, the increased serum lipids levels and reduced adiponectin levels also confirmed the metabolic abnormalities caused by fructose. As expected, berberine significantly improved insulin sensitivity and reversed these metabolic abnormalities in the fructose group. These results confirmed the protective effects of berberine against fructose-induced insulin resistance, which are in agreement with previous reports (Gao et al. 2008; Yue et al. 2019).

Fructose has previously been reported to increase hunger and stimulate the appetite (Page et al. 2013; Lowette et al. 2015). To investigate whether the metabolic consequences caused by fructose were a result of excess food intake, we measured the body weight and food consumption weekly. We found that fructose inhibited the appetite from week 5 to week 6, whereas this inhibitory effect gradually disappeared after week 6. Leptin and orexin play a crucial role in controlling appetite. Numerous studies have demonstrated that chronic fructose consumption stimulates the over-secretion of leptin and finally leads to leptin resistance (Shapiro et al. 2008; Bursac et al. 2014). However, the time-effect of fructose-induced leptin resistance has rarely been reported. In the present study, the reduced appetite might be attributed to the increased leptin secretion stimulated by fructose. After week 6, the appetite and body weight were restored in fructose-fed mice, which suggested the occurrence of leptin resistance induced by fructose. This speculation was verified by the increased serum levels of orexin and leptin in the fructose-fed mice. Berberine reversed the changes of leptin and orexin. Although berberine did not affect the body weight, it reduced the fat/body weight ratio.

Insulin signalling plays pivotal roles in maintaining metabolic homeostasis by promoting glycogen storage and inhibiting lipolysis. In our previous report, fructose impaired insulin signalling by decreasing the phosphorylation expression of AKT, insulin receptor (IR) and insulin receptor substrates 1 (IRS-1; Li et al. 2010). As a key molecule of the insulin signalling pathway, AKT can promote glycogen synthesis by phosphorylating and inactivating GSK3β. We verified that fructose increased the TG content and reduced the glycogen content in the liver, which were reversed by berberine. Moreover, we also found that berberine upregulated the phosphorylation levels of AKT and GSK3β, which confirmed the improvement of insulin signalling. Previous reports have shown that reduced PGC1α disrupts hepatic insulin signalling in the mouse (Estall et al. 2009; Besse-Patin et al. 2017). A recent study confirmed that PGC1α is involved in insulin-stimulated AKT phosphorylation (Besse-Patin et al. 2019). PGC1α could increase AKT and p-AKT expression in muscle (Romanino et al. 2011). In the present study, we confirmed that hepatic PGC1α expression was downregulated in the fructose-fed group, which was reversed by pioglitazone and berberine treatment. Since PGC1α is an important transcriptional coactivator that stimulates mitochondrial biogenesis and regulates energy metabolism (Handschin and Spiegelman 2006), our results suggested that the protective effect of berberine against insulin resistance might be related to the upregulation of PGC1α.

AMPK has been extensively investigated as a potential therapeutic target of insulin resistance (Ruderman et al. 2013). Activated AMPK phosphorylates and activates the insulin receptor, directly enhancing the insulin signalling pathway (Chopra et al. 2012). Treatment of mice with β-guanidinopropionic acid, an AMPK activator, increased PGC1α expression and mitochondrial density in muscle (Zong et al. 2002). Fructose damaged hepatic AMPK signalling and lead to insulin resistance (Gugliucci 2016). In the present study, we found that fructose significantly decreased the phosphorylation level of AMPK and increased the AMP/ATP ratio. These results confirmed that fructose accelerated ATP consumption, partially through promoting lipids synthesis. It is a paradox that fructose increased AMP levels, but inhibited AMPK activation. AMP is an allosteric activator of AMPK that acts by binding to the γ-subunits of AMPK; however, the phosphorylation activation of AMPK relies on two main upstream kinases, LKB1 and CaMKKβ (Sriwijitkamol et al. 2006; Willows et al. 2017). To explore the underlying mechanism, we examined the expression of LKB1 and CaMKKβ. We found that fructose decreased the expression of CaMKKβ. Therefore, the increased AMP levels did not activate AMPK phosphorylation, which could be attributed to the inhibition of CaMKKβ caused by fructose. Both pioglitazone and berberine elevated AMPK phosphorylation levels, promoted ATP production, and decreased the AMP/ATP ratio. Importantly, we found that pioglitazone significantly increased the expression of CaMKKβ whereas berberine increased the phosphorylation levels of LKB1. These results suggested that berberine activates AMPK mainly though LKB1.

## Conclusions

The present study provides experimental evidence of the protective effects of berberine against insulin resistance induced by chronic fructose consumption. Moreover, our results suggested that this favourable effect could be attributed to activation of the LKB1/AMPK/PGC1α pathway. Our findings may provide an experimental basis for the application of berberine in the treatment of insulin resistance.
